# Supplementary material for: Polymeric Micelles Formulation of Combretastatin Derivatives with Enhanced Solubility, Cytostatic Activity and Selectivity against Cancer Cells
Source: Pharmaceutics. 2023 May 29;15(6):1613. doi: 10.3390/pharmaceutics15061613 (PMC10304518; doi:10.3390/pharmaceutics15061613)

Polymeric micelles formulation of combretastatin derivatives with enhanced solubility, cytostatic activity and selectivity against cancer cells

Igor D. Zlotnikov [1], Artem S. Ferberg [1], Alexander A. Ezhov [2], Sergey S. Krylov [3], Marina N. Semenova [4], Victor V. Semenov [4] and Elena V. Kudryashova [1,*]

[1] Faculty of Chemistry, Lomonosov Moscow State University, Leninskie Gory, 1/3, 119991 Moscow, Russia; zlotnikovid@my.msu.ru (I.D.Z.);
[2] Faculty of Physics, Lomonosov Moscow State University, Leninskie Gory, 1/2, 119991 Moscow, Russia; alexander-ezhov@yandex.ru
[3] N. D. Zelinsky Institute of Organic Chemistry RAS, 47 Leninsky Prospect, 119991 Moscow, Russia; forvard1953@yandex.ru (S.S.K.); vs@chemical-block.com (V.V.S.)
[4] N. K. Koltzov Institute of Developmental Biology RAS, 26 Vavilov Street, 119334 Moscow, Russia; ms@chemical-block.com

* Correspondence: helena_koudriachova@hotmail.com (E.V.K.)

Content

Video S1. NTA video of extruded Chit5-OA-20 micelles. T = 25 °C. MilliQ H_2O. 10^9-10^{10} particles/mL. Avanti Polar Lipids mini-extruder was used with 400 nm membrane.

Figure S1. The scheme of synthesis of grafted chitosans. m = 5 – percentage of acylated pyranose fragments, n = 95 – percentage of deacylated pyranose fragments, a – experimental modification degree. Reprinted from article [Zlotnikov, 2023].

Figure S2. Parameters of the sources used for excitation and registration of fluorescence in optical microscopy of cells.

Figure S3. ^{1}H NMR of (a) Chit5-OA-20 non-loaded and loaded with pyrazole **1** and triazole **2**; (b) Chit5, (c) Chit5-MUA-20, (d) Chit5-LA-20. D_2O. T = 25 °C.

Figure S4. Micrographs visualizing the solubility of cytostatics in micellar form and precipitation from DMSO when diluted with buffer. The width of the photos is 230 nm.

Figure S5. Fluorescence emission spectra of albendazole **3** and pyrazole **1**. λ_{exci}(albendazole **3**) = 280 nm, λ_{exci}(pyrazole **1**) = 260 nm. T = 22 °C.

Figure S6. FTIR spectra of Chit5-MUA-20 loaded with albendazole **3**. D_2O. 22 °C

Figure S1. The scheme of synthesis of grafted chitosans. m = 5 – percentage of acylated pyranose fragments, n = 95 – percentage of deacylated pyranose fragments, a – experimental modification degree. Reprinted from article [Zlotnikov, 2023].

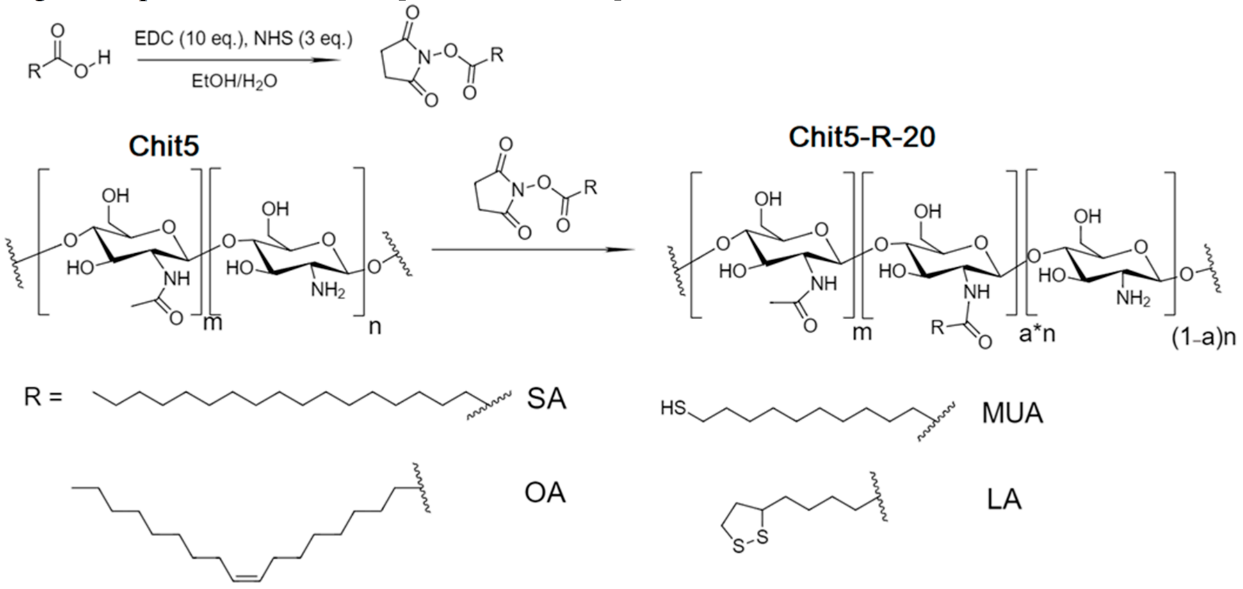

Figure S2. Parameters of the sources used for excitation and registration of fluorescence in optical microscopy of cells.

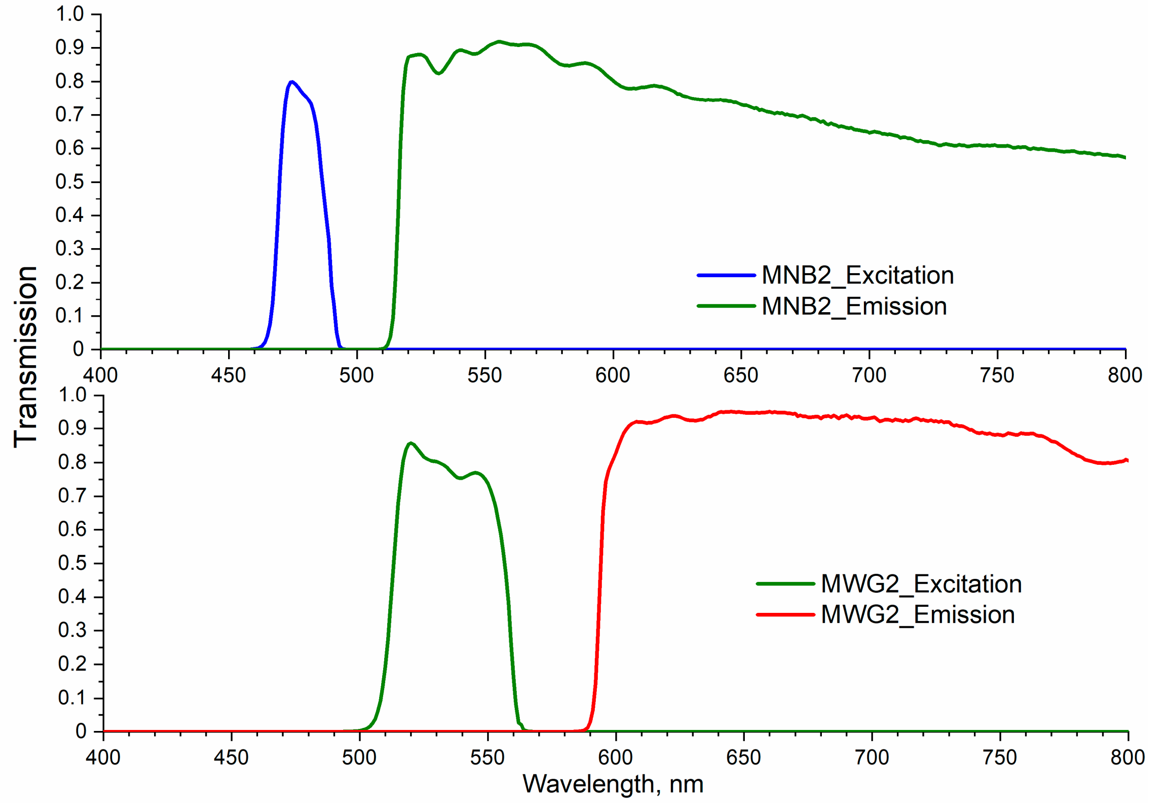

Figure S3. ^{1}H NMR of (a) Chit5-OA-20 non-loaded and loaded with pyrazole **1** and triazole **2**; (b) Chit5, (c) Chit5-MUA-20, (d) Chit5-LA-20. D_2O. T = 25 °C.

(a)

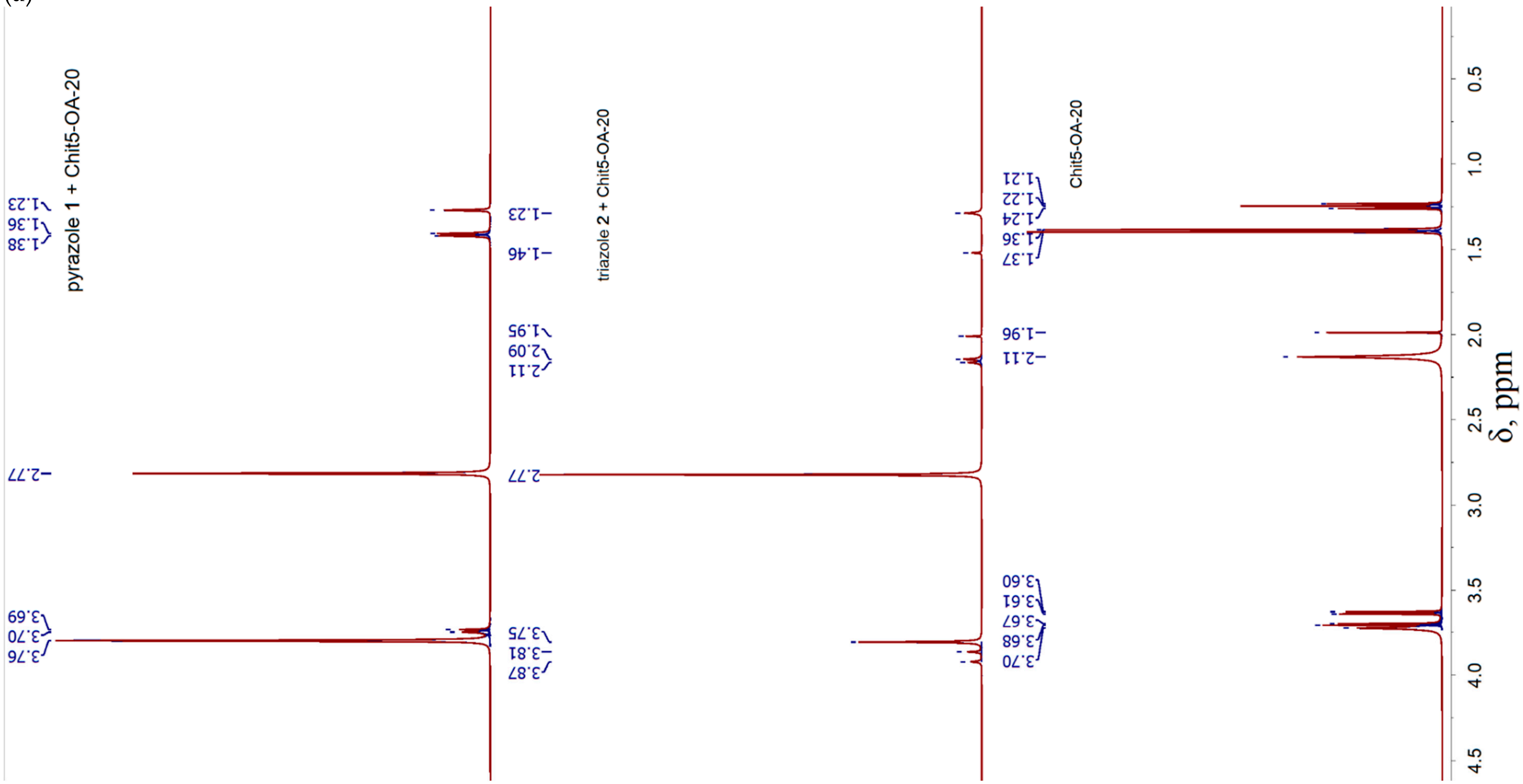

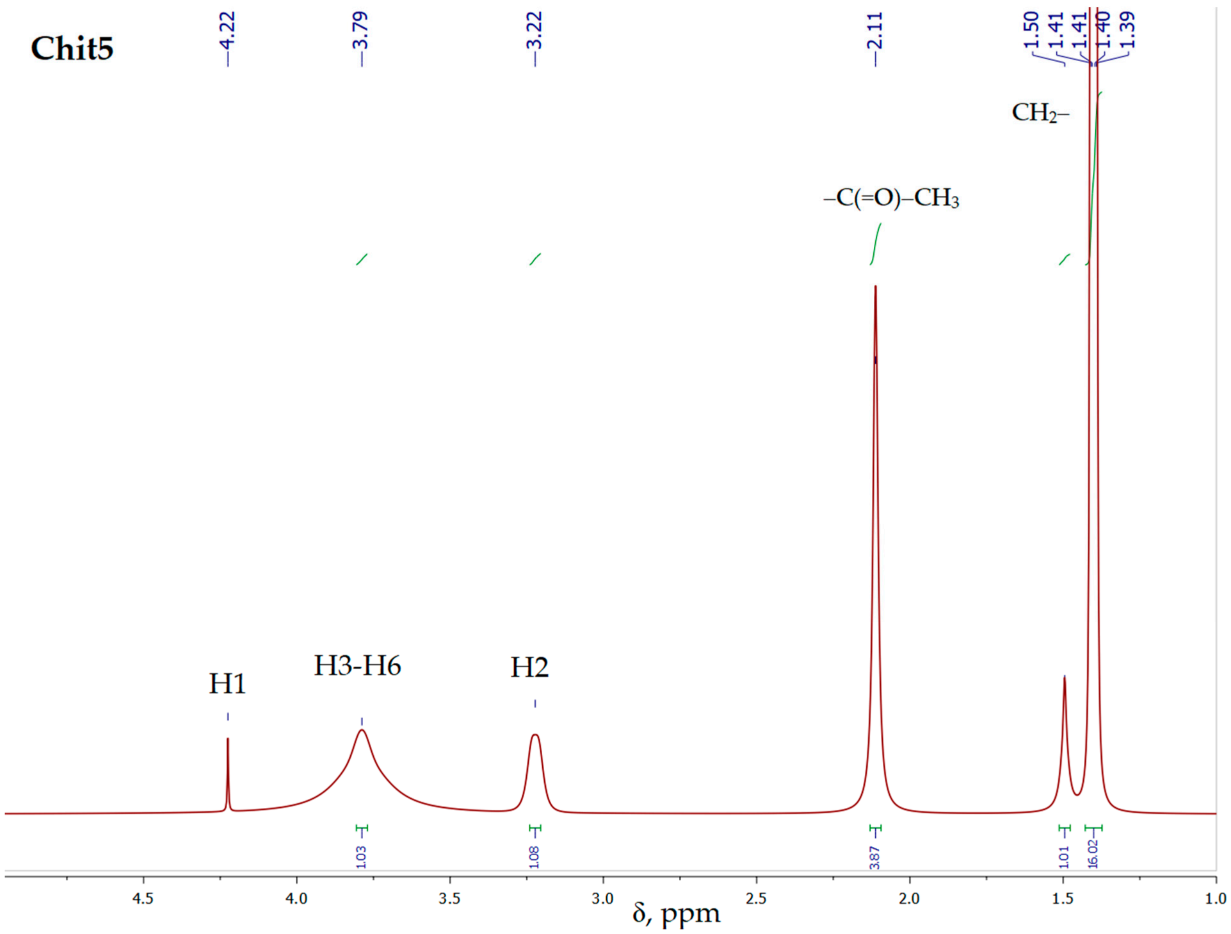
Chit5
4.22
3.79
3.22
2.11
1.50
1.41
1.41
1.40
1.39
CH2–
–C(=O)–CH3
H1
H3-H6
H2
1.03
1.08
3.87
1.01
16.02
4.5
4.0
3.5
3.0
2.5
2.0
1.5
1.0
δ, ppm

(b)

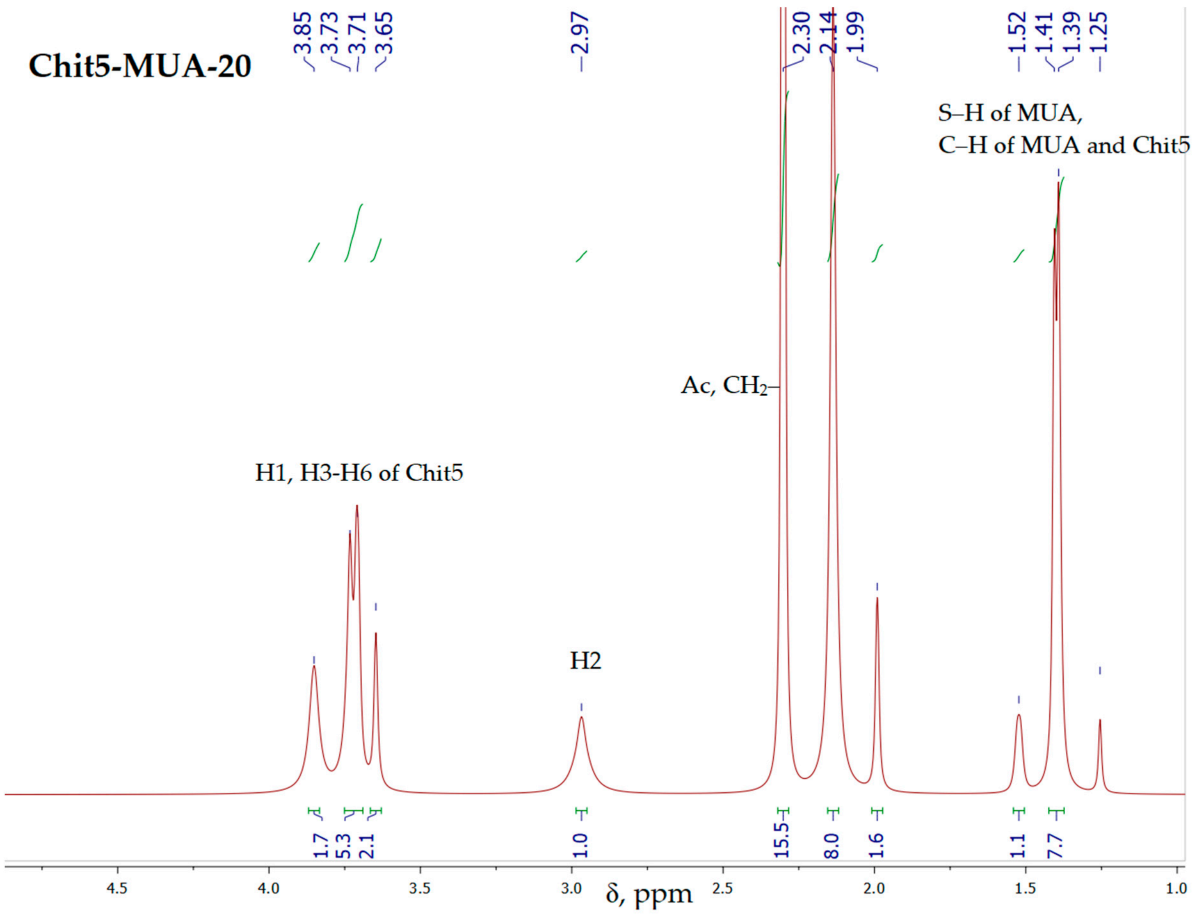

Chit5-MUA-20
3.85
3.73
3.71
3.65
2.97
2.30
2.14
1.99
1.52
1.41
1.39
1.25
S–H of MUA,
C–H of MUA and Chit5
Ac, CH2–
H1, H3-H6 of Chit5
H2
1.7
5.3
2.1
1.0
15.5
8.0
1.6
1.1
7.7
4.5
4.0
3.5
3.0
δ, ppm
2.5
2.0
1.5
1.0

(c)

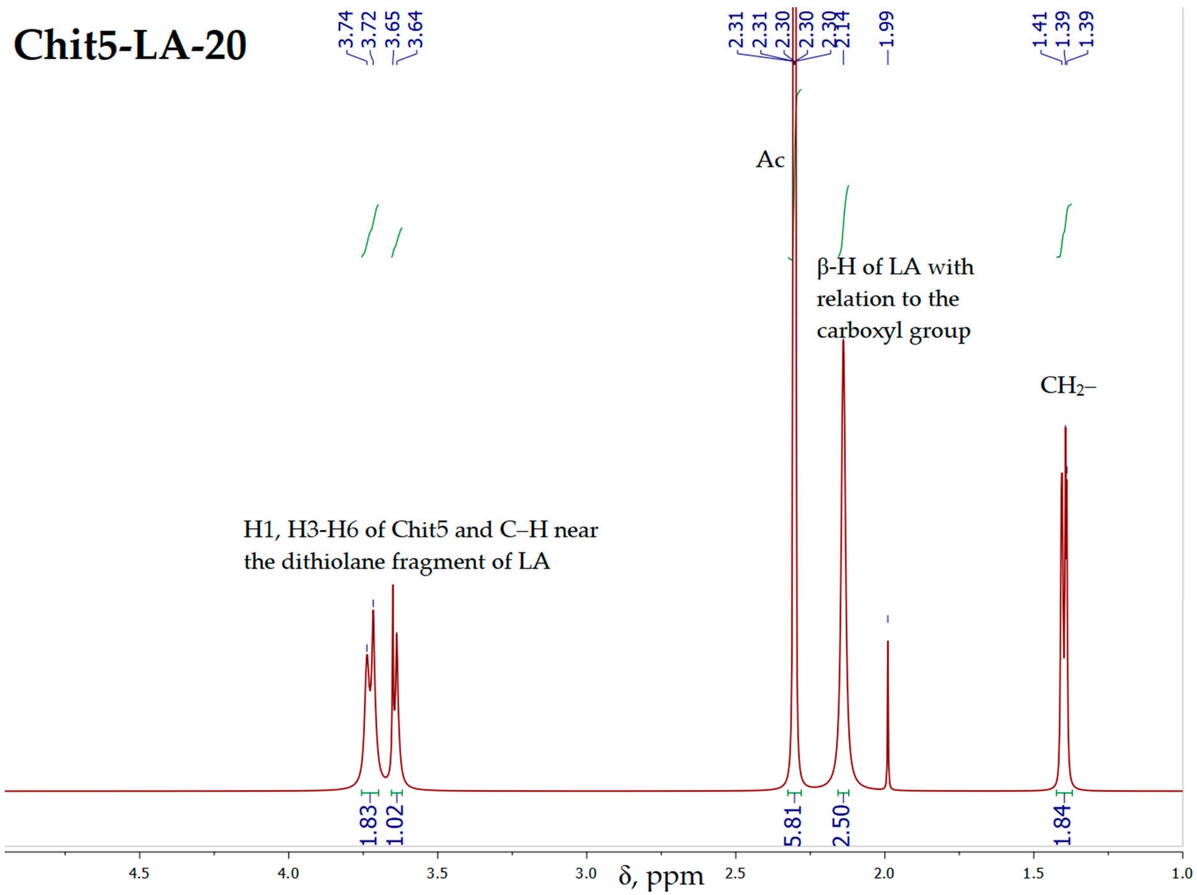

Chit5-LA-20
3.74
3.72
3.65
3.64
2.31
2.31
2.30
2.30
2.30
2.14
1.99
1.41
1.39
1.39
Ac
β-H of LA with relation to the carboxyl group
CH2–
H1, H3-H6 of Chit5 and C–H near the dithiolane fragment of LA
1.83
1.02
5.81
2.50
1.84
4.5
4.0
3.5
3.0
δ, ppm
2.5
2.0
1.5
1.0

(d)

Figure S4. Micrographs visualizing the solubility of cytostatics in micellar form and precipitation from DMSO when diluted with buffer. The width of the photos is 230 nm.

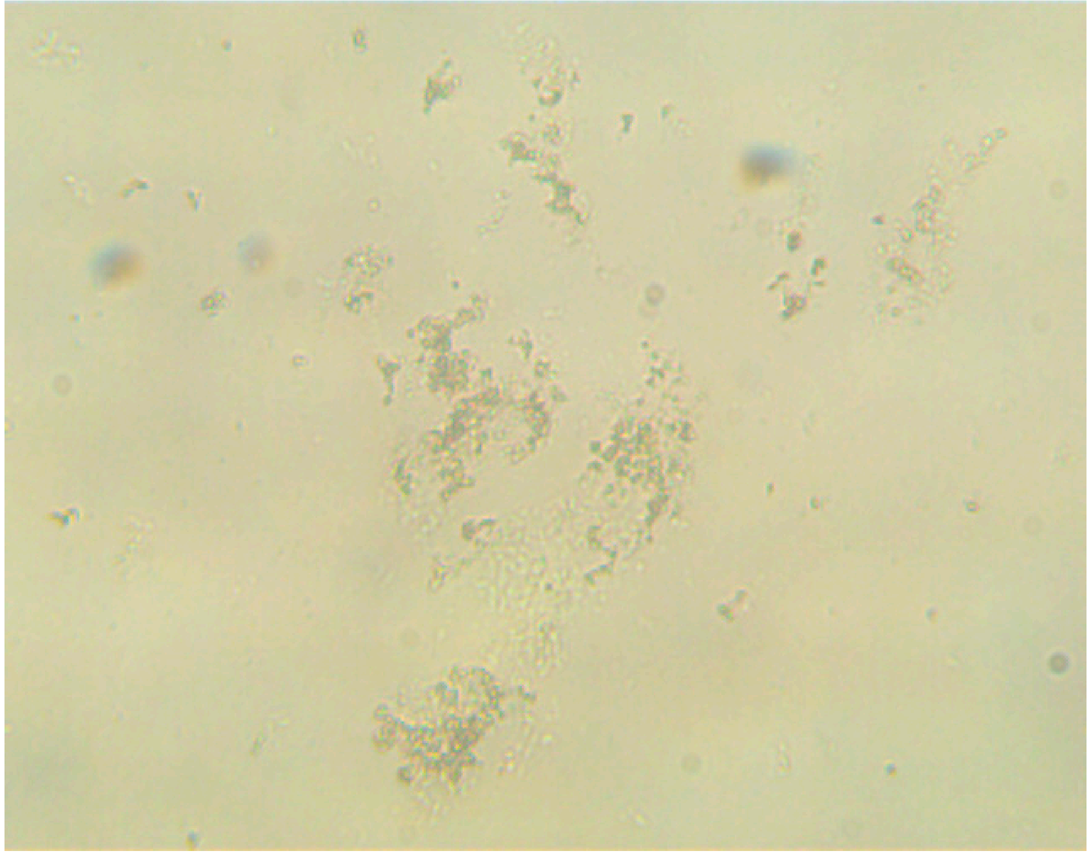

(a) Pyrazole **1** dropped out of DMSO when diluting PBS

(b) Pyrazole **1** partially insoluble in a shortage of micelles (5 to 1 w/w)

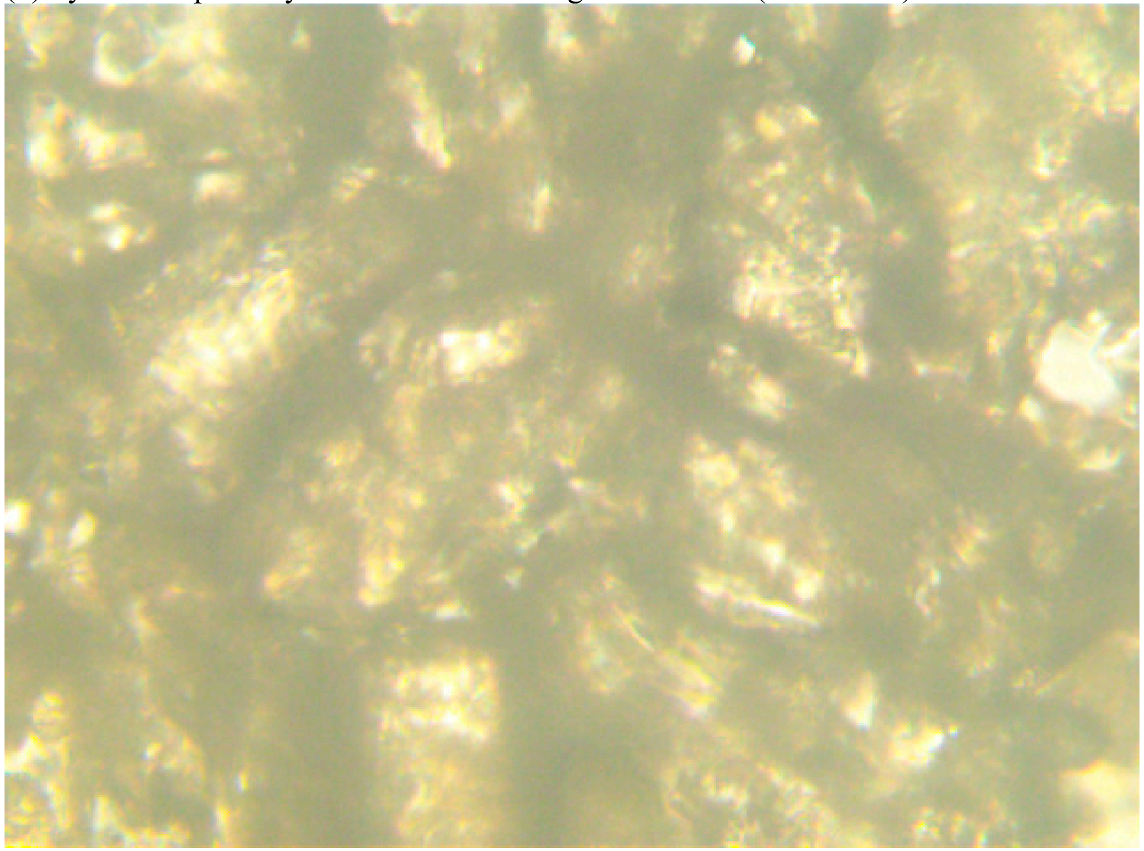

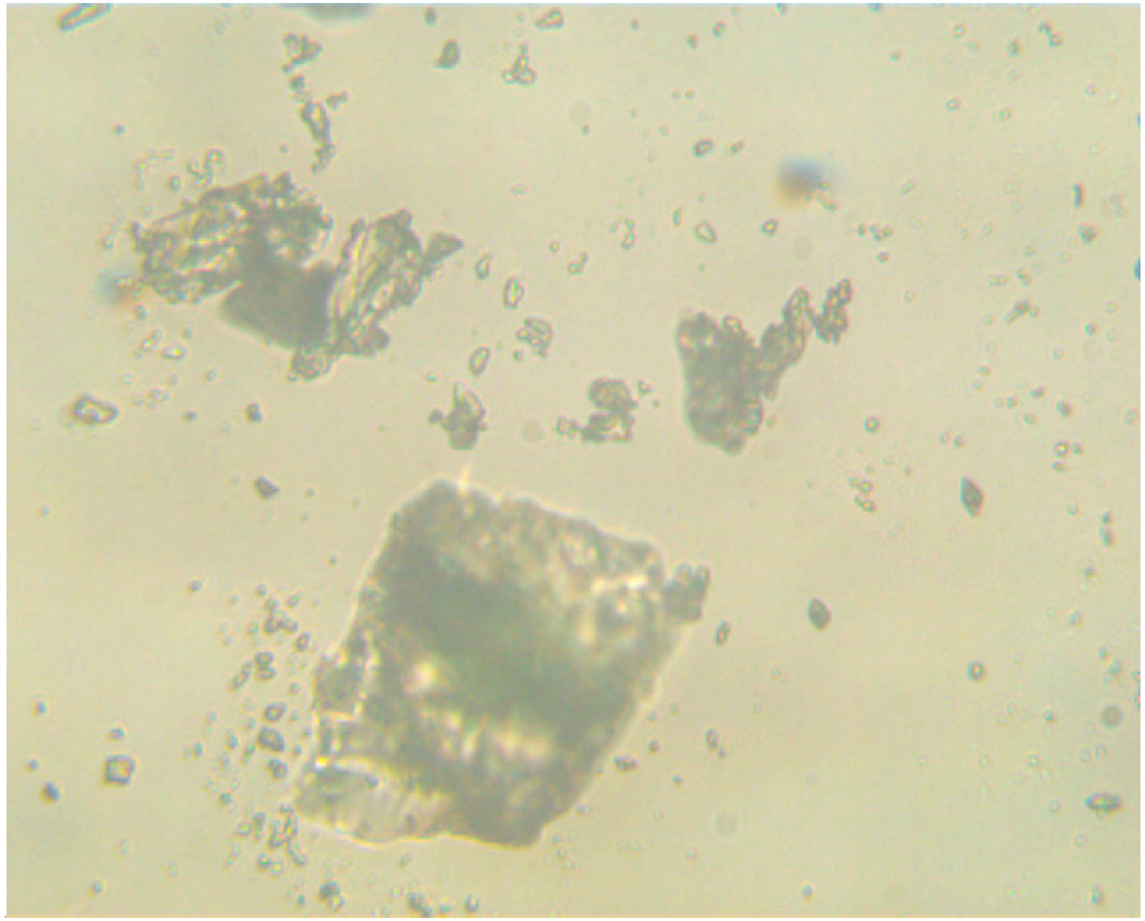

(c) Triazole **2** dropped out of DMSO when diluting PBS

(d) Diarylisoxazole **4** dropped out of DMSO when diluting PBS

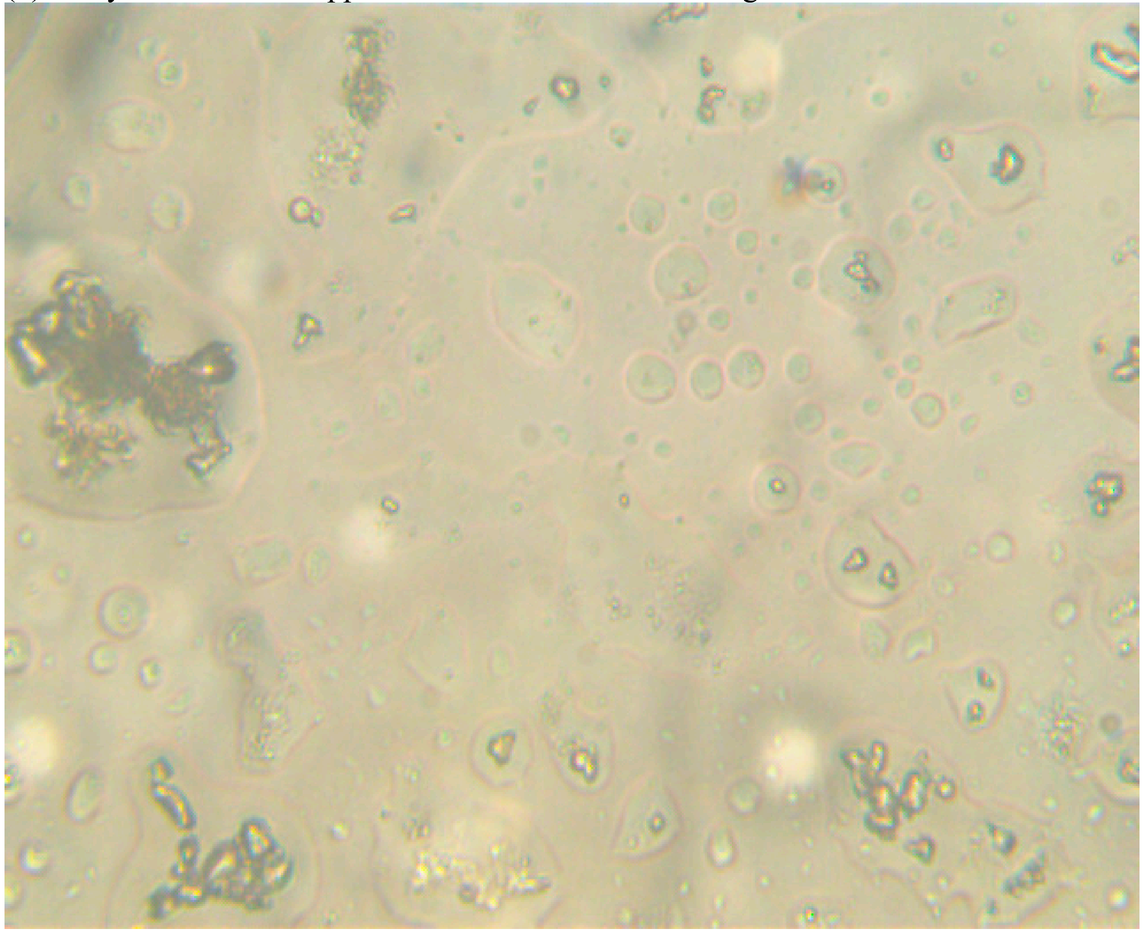

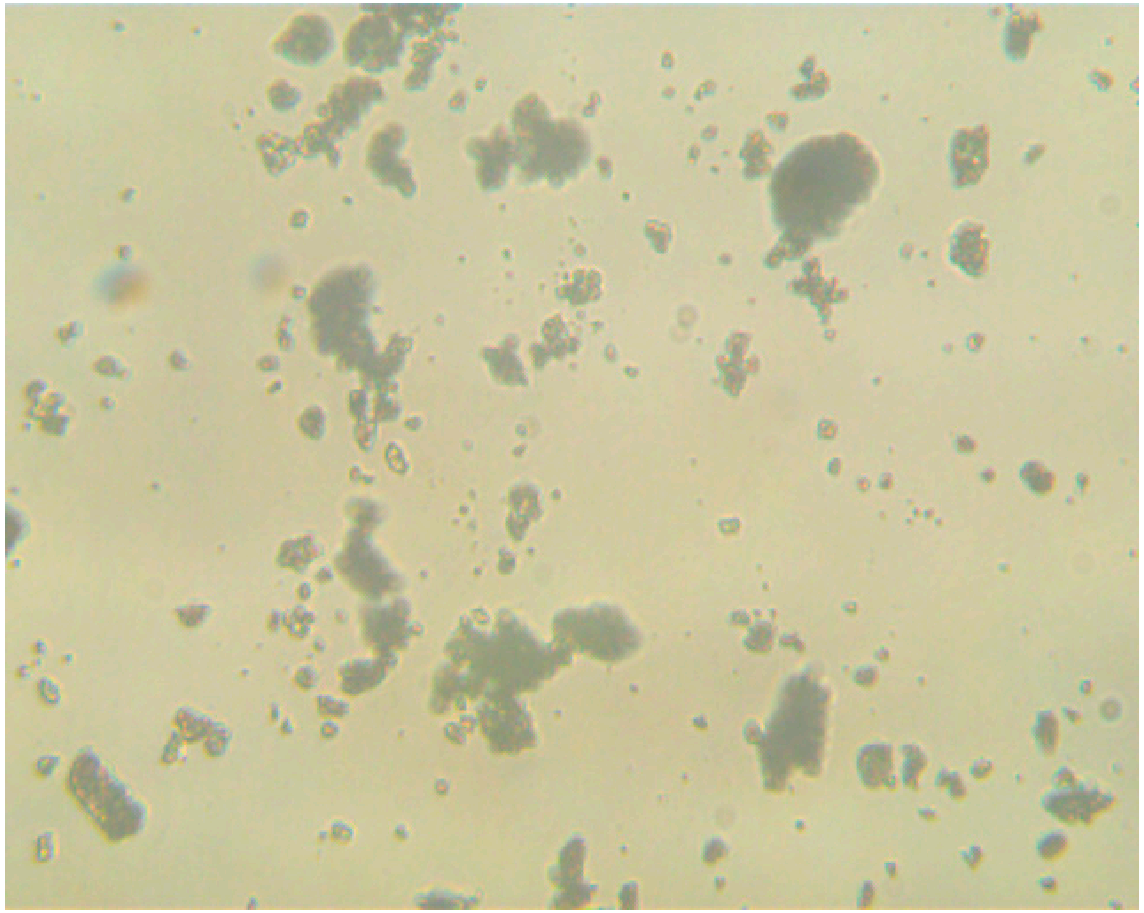

(e) Albendazole **3** dropped out of DMSO when diluting PBS

(f) Albendazole **3** dissolved in micelles 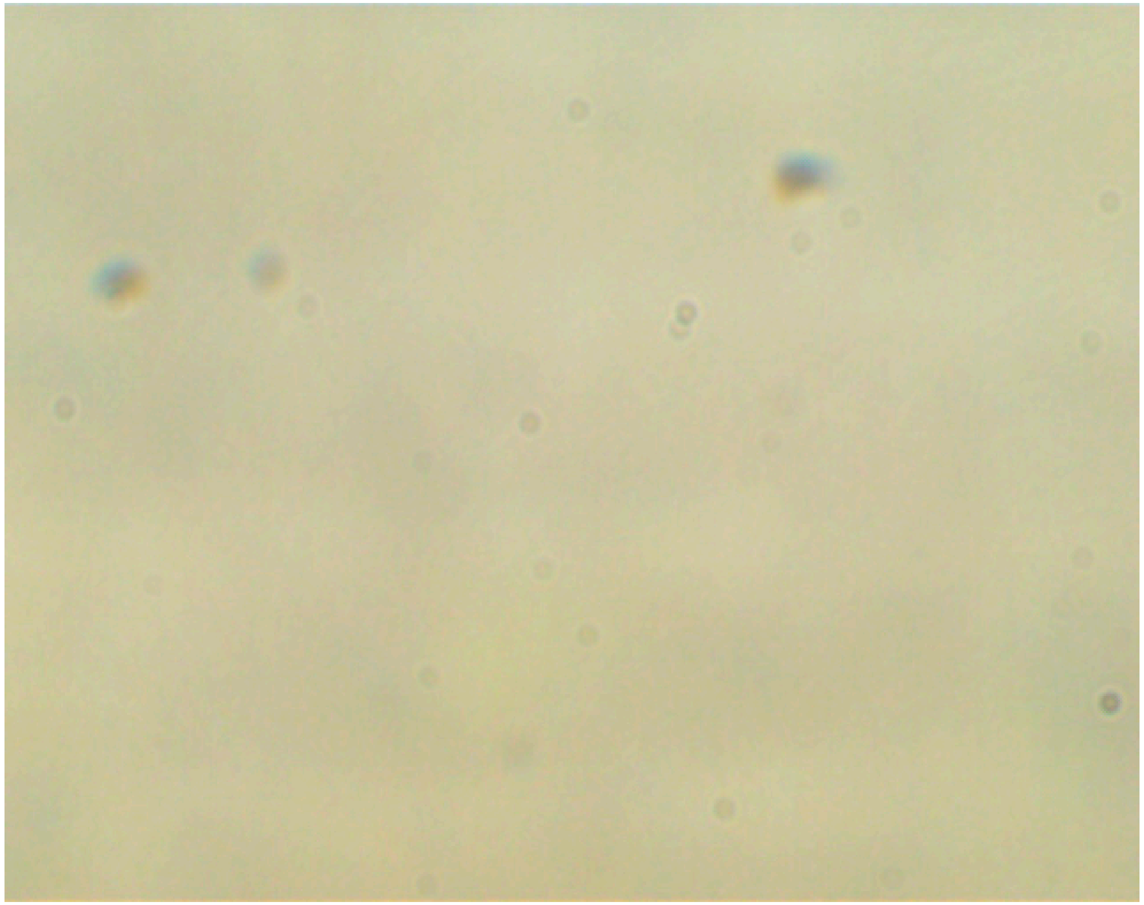

Figure S5. Fluorescence emission spectra of albendazole **3** and pyrazole **1**. λ_{exci}(albendazole **3**) = 280 nm, λ_{exci}(pyrazole **1**) = 260 nm. T = 22 °C.

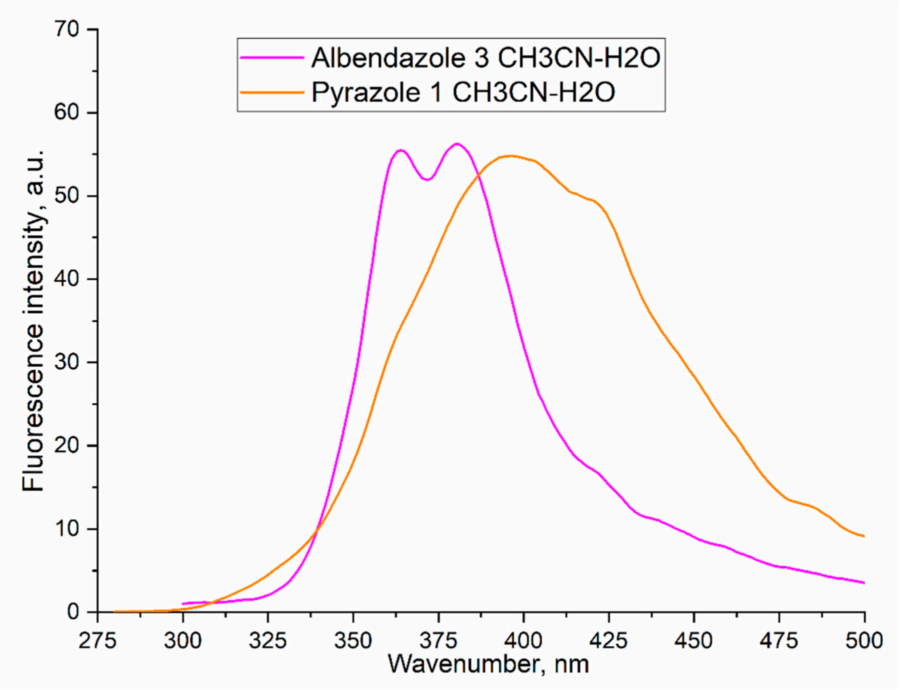

Figure S6. FTIR spectra of Chit5-MUA-20 loaded with albendazole **3**. D_2O. 22 °C

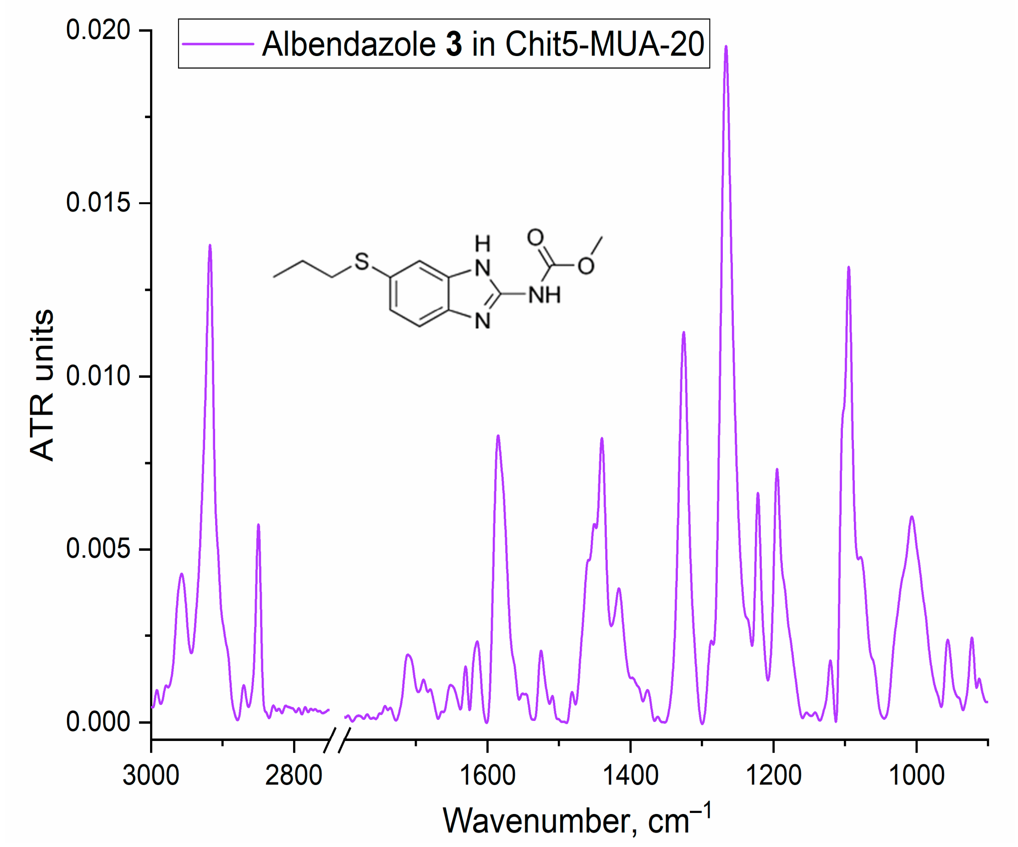

Supplement: Supplementary file 1 [file pharmaceutics-15-01613-s001.zip › pharmaceutics-2350180-supplementary.pdf]
